# Uronium peptide coupling agents: Another case of occupational airborne allergic sensitization induced by HBTU

**DOI:** 10.1002/psc.3649

**Published:** 2024-08-09

**Authors:** Valentina Borghesani

**Affiliations:** ^1^ Department of Chemistry, Life Sciences, and Environmental Sustainability University of Parma Parma Italy

**Keywords:** allergic reaction, coupling agents, occupational allergic sensitization, peptide synthesis

## Abstract

Uronium peptide coupling agents (HBTU, HATU, and HCTU) create a special hazard as they are immune sensitizers. Few reported cases are mentioned in the literature; despite that, it is important to raise the awareness on the subject and to highlight the risk and potential symptoms that could occur to those who directly work in contact with uronium peptide coupling agents, as well as to the safety deputies in the universities and industries. Based on a personal experience, the health impact of laboratory exposure to HBTU is described, and the insights gained from the experience are developed. A skin irritation reaction and allergy symptoms induced by HBTU exposure are shown here as well as the rate of worsening of symptoms since the first allergic reaction. Recommendations for handling coupling agents more safely in the research laboratory will also be given, and a casuistry of the matter to help other lab‐users to recognize, assess, minimize, prepare for emergencies (RAMP) process.

## Graphical abstract

The health impact of laboratory exposure to HBTU is described based on a case study, showing the skin irritation reaction and allergy symptoms induced and the rate of worsening of symptoms since the first allergic reaction. Recommendations for handling coupling agents more safely in the research laboratory are given.

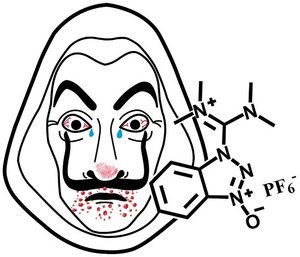

## 1 INTRODUCTION

**FIGURE 1:**
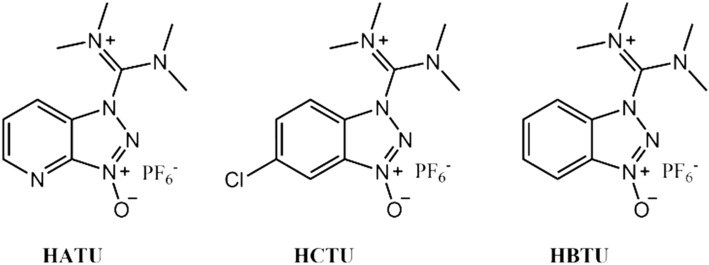
Chemical structures of the uronium coupling agents HATU (1‐[bis[dimethylamino]methylene]‐1*H*‐1,2,3‐triazolo[4,5‐*b*]pyridinium‐3‐oxide hexafluorophosphate), HCTU (*O*‐[1*H*‐6‐chlorobenzotriazole‐1‐yl]‐1,1,3,3‐tetramethyluronium hexafluorophosphate), and HBTU (2‐[1*H*‐benzotriazole‐1‐yl]‐1,1,3,3‐tetramethyluronium hexafluorophosphate).

Uronium peptide coupling agents HBTU, HATU, and HCTU (Figure 1) are the most common coupling agents used for peptide synthesis. As their names suggest, these reagents were initially believed to have a uronium structure, but crystal and solution structure studies revealed that these reagents actually have an aminium structure.^1^, ^2^

HBTU, along with its analogue TBTU (which has BF_4_^−^ as the counterion instead of PF_6_^−^), is a very efficient peptide coupling reagent and causes minimal racemization. Coupling reactions are quick, and to reduce racemization to insignificant levels, hydroxybenzotriazole (HOBt) can be added. This makes these reagents the choice for critical applications, and they are widely used in peptide synthesis by the majority of the scientific community. HATU is similar to HBTU, but although it reacts faster with less epimerization during coupling, if used in excess, it can react with the unprotected N‐terminal and block further chain elongation.

All these coupling agents are widely used by many people for synthesis, and most users do not develop any allergic reactions, even after years of use.

This paper aims to transform (another) airborne allergic reactions into a new awareness for the laboratory community, as well as for health and safety professionals, and to discuss an alternative peptide coupling strategy.

There are only a few cases reported in the literature, and the Safety Offices of Universities or Industries are often not up to date on the risks associated with the use of these reagents.

In 2020, McKnelly et al. reported a case of anaphylaxis following long‐term exposure to uronium coupling agents^2^ (HATU, HBTU, and HCTU).^3^ A 27‐year‐old female researcher (F‐Res) developed life‐threatening anaphylaxis after few years of working with these agents. Initially, after 1 year of exposure, she experienced allergy symptoms such as sneezing, coughing, and a runny nose, which gradually progressed to anaphylaxis.^3^

This case is the latest in a series of reported occupational airborne allergic sensitizations, yet the potential risks associated with these reagents remain underestimated. Despite the limited literature on the subject, the Material Safety Data Sheets (MSDS) for HBTU, HATU, and HCTU have only recently started to include warnings about possible allergic reactions. The MSDS for these agents now feature, in Section 2—Label elements, the hazard statement H317, “May cause an allergic skin reaction,” and for HATU, H334, “May cause allergy or asthma symptoms or breathing difficulties if inhaled.” However, the MSDS for HBTU in Section 11 (Toxicological Information) reports no skin irritation following the OECD Test Guideline 439 and no eye irritation based on tests conducted on bovine corneas (OECD Test Guideline 437).^4^

Despite these updates, uronium coupling agents are insidious because severe allergies can develop slowly over several years, varying by individual, until they become life‐threatening. Once sensitized, even minimal exposures can trigger symptoms. Additionally, sensitized individuals may exhibit symptoms through secondary exposure from others who handle these reagents, potentially rendering them unable to work with or around these compounds.

A significant issue with interpreting MSDSs is understanding the term “sensitizer.” While terms like “explosive,” “flammable,” or “carcinogenic” clearly indicate how to handle a reagent to prevent accidents, “sensitizer” is more nebulous, identifying substances that can potentially become allergens. This ambiguity leads many lab workers to underestimate the risk, thinking “it can happen, but not to me.” There is no comprehensive list of chemicals that will act as sensitizers or predict who might be vulnerable to them.^5^

Chemical sensitizers fall into two categories: those that induce respiratory symptoms and the more common type that affects the skin. Plackett in 2020^5^ highlights another underestimated aspect of sensitization: the latency period. Since antibody formation takes time, allergic responses may not occur upon first exposure but can manifest unexpectedly, from 10 days to many years later. Typically, symptoms appear within the first 2 years of exposure.^5^

## 2 ALLERGIC CASE

**FIGURE 2:**
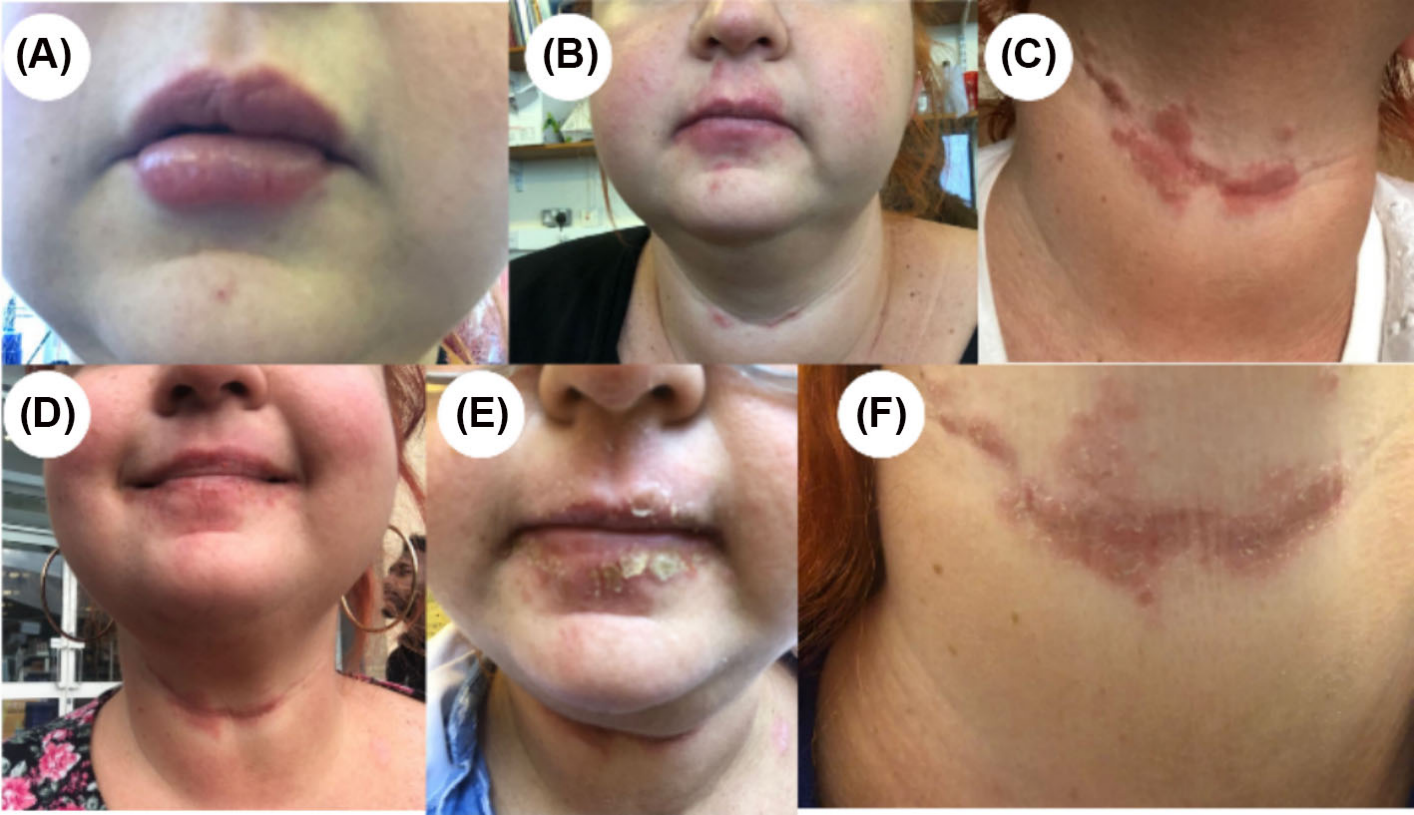
Allergy symptoms developed in 2021 by female researcher (F‐Res) when weighing out HBTU and/or dissolving it under the fume hood. (A) The first brush with allergy‐induced lip swelling less than an hour of exposure to HBTU. (B,C) Typical skin rushes appeared around her mouth and on her cheeks (B) and in the neck crease (C) starting from the second allergic event. (D–F) The progression of skin rushes 1 week after exposure.

Here, we report a case of a 31‐year‐old F‐Res who developed a severe skin irritation. This irritation initially presented as reddened skin but quickly progressed to a rash with irritated, swollen skin, and itchy, painful lesions (Figure 2).

In the second half of 2019, F‐Res joined a group working with metallopeptides. After 6 months, she noticed a mild skin irritation around her mouth (Figure 2A,B) but did not associate it with the use of HBTU. About 3 months later, after returning to the laboratory following the lockdown, she resumed synthesizing various peptides. In the following months, she began extensively using the uronium peptide coupling agent HBTU. Almost immediately, F‐Res started to develop skin irritation, which became progressively more severe with each exposure (Figure 2). The severity of the skin injuries increased with each exposure, while the time required to trigger the allergic reaction decreased.

The allergic symptoms resembled allergic eczema, which can appear similar to burns. The skin became itchy, scaly, or raw. F‐Res exhibited blisters that wept, oozed, or became crusty. Additionally, the skin on her face appeared dry, rough, flaky, inflamed, irritated, and itchy.

**FIGURE 3:**
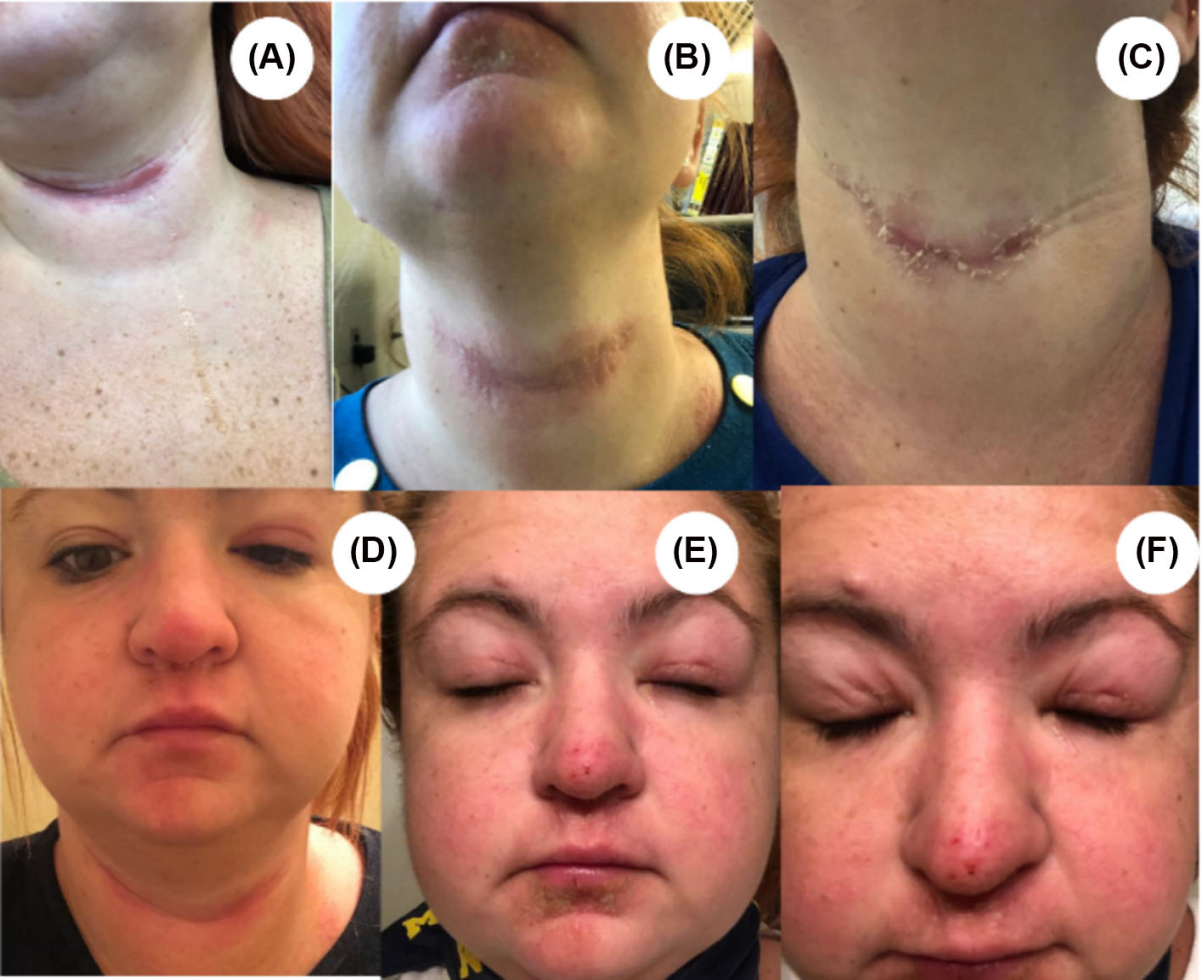
Allergy symptoms developed in 2022 by female researcher (F‐Res) when exposed to HBTU solution. (A–C) Lesions appeared on her neck crease less than 1 day after exposure (A,B) and after 10 days (C). (D–F) Allergically induced eye swelling after 12 h (D) and progressed over 3 days (E,F).

Given the limited information found on the MSDSs in 2021, F‐Res concluded that the allergen was HBTU by process of elimination. She observed no allergy symptoms when weighing out Fmoc‐protected amino acids or preparing the deprotection solution (20% piperidine in DMF). However, she experienced almost immediate itching around her mouth and neck when weighing out HBTU. In September 2019, she suspected developing an allergy to coupling agents. In fact, a few hours after manipulate HBTU powder, the skin on her face became sore. After 8–10 h, her eyes were swollen, and she had lesions around her mouth and neck crease (Figure 3). To minimize exposure, all precautions suggested by McKnelly et al.^3^ were taken: weighing out HBTU powder under a fume hood, wearing a respiratory mask and face shield, disposing of contaminated waste in a closed bin, and changing gloves immediately after use. Despite these measures, airborne particulate matter still caused allergic reactions.

**FIGURE 4:**
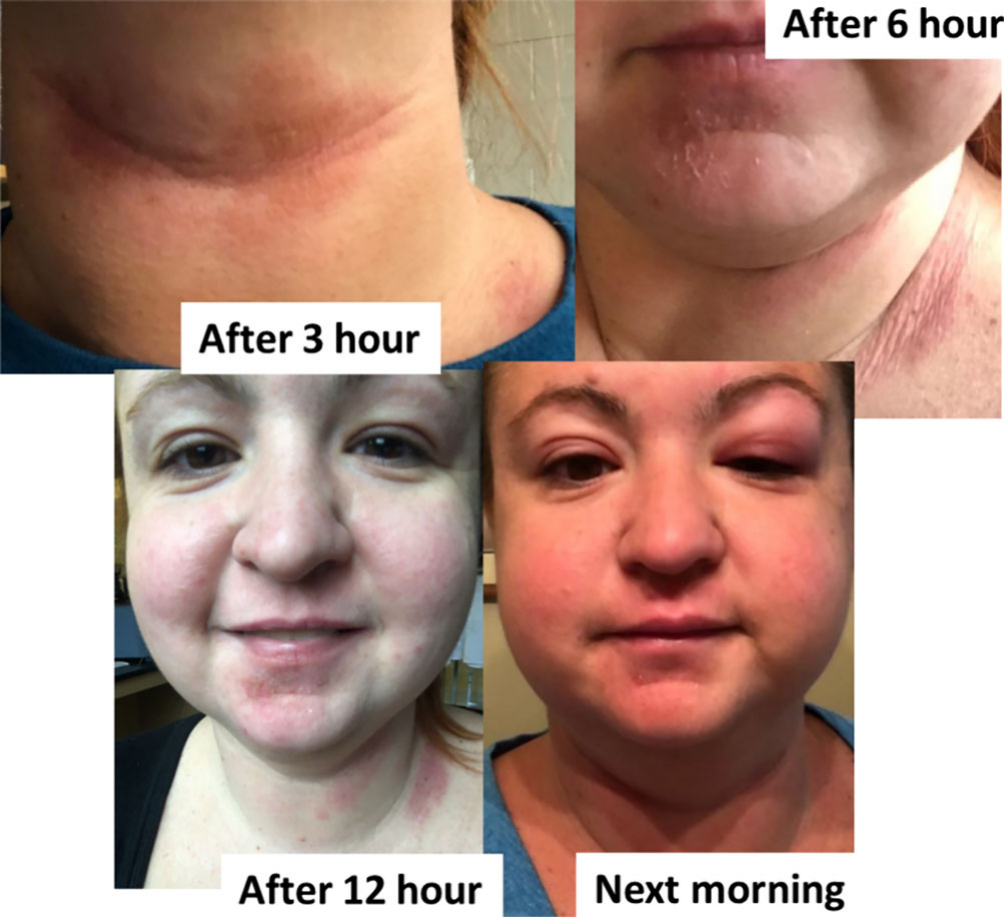
Allergy symptoms developed during the next 24 h after exposure in 2023 by female researcher (F‐Res) only by entering in the laboratory where a coworker was using HBTU under the fume hood.

The situation worsened month by month, eventually causing allergy symptoms even when she merely entered the laboratory where someone had used the reagent or where an open bottle containing HBTU solution was present (Figure 4).

To mitigate her allergy symptoms, F‐Res had to take antihistamines such as diphenhydramine (generic Benadryl) and cetirizine HCl 10 mg (generic Zyrtec). For quicker recovery (1 week instead of 2–3 weeks) from itching, skin lesions, and swelling of the eyes and nose, the hospital prescribed her also cortisone (methylprednisolone, generic Medrol).

Although there is no clinical evidence correlating her exposure to HBTU with the partial loss of smell she reported, F‐Res suspects a connection. During her last use of HBTU as a peptide coupling agent, she experienced the usual allergic reactions along with swelling and irritation of her nose and upper respiratory tract. After these episodes, she noticed a diminished ability to smell, particularly the thiols in the laboratory.

From 2006 to 2010, six more instances of chemical sensitization from HBTU were reported with similar respiratory and skin reactions,^3^, ^6^, ^7^, ^8^ and nine cases in total have been documented since 2003 (Table 1).^5^, ^9^, ^10^, ^11^

**TABLE 1:** Reported allergic symptoms since 2003 induced by uronium coupling peptide agents use.

Sensitizer	Symptoms		Duration of exposure before onset of symptoms	Researcher's gender and age	Medicament prescribed	Ref.	When reported?
HBTU	Skin irritation (very red, sore, and itchy periocular and perioral areas of the face and on the neck also with lesions and vesicles)		1.5 year	Female; 31	Antihistamines cetirizine (generic Zyrtec) or antihistamine diphenhydramine (generic Benadryl) and methylprednisolone (cortisone)	This work	2021–2023
HATU HBTU HCTU	Anaphylaxis Initially: sneezing and runny nose (using HCTU); 3 months later: wheezing slightly. 1 month later: cough, sneeze, feeling of tightness in the throat, and wheezing		10 months (1st allergy symptoms) +2 years (progressed to the point of anaphylaxis)	Female; 27	Antihistamine diphenhydramine (generic Benadryl) Do not access to building where coupling agents are used and carry an epinephrine autoinjector (generic EpiPen) as a safety precaution.	^3^	2020
HBTU	Hand dermatitis. On one occasion also involved the periocular and perioral areas of the face, the neck, and the leg.		14 years	Female; 37	Topical steroids	^6^	2010
TBTU HBTU	Respiratory (rhinitis and/or asthma) and skin hypersensitivity reactions (transient urticaria rash of the face and hands and persistent eczematous‐like skin lesions on the hands)	Rhinitis + asthma	2 years and 7 months	Female; 28	No medication Improvement in the plant ventilation system, isolation of the weighing procedure, use of protective masks, and long‐sleeved gloves	^7^	2008
		Skin + asthma (allergy only to TBTU)	3 months	Male; 30			
		Skin + rhinitis	8 months	Male; 25			
		Skin + rhinitis	2 months	Female; 24			
HBTU	Initially: redness and burning sensation on his face associated with dyspnea and faintness 6 months later: redness on the face, cough, and dyspnea. Feeling dizzy. Shallow respiration and urticaria on the face and flexural areas on extremities was observed. The palms of hands were also swollen.		3 years	Male; 28	Oral antihistamine Intramuscular corticosteroid	^8^	2006
HBTU	Dermatitis, initially limited to hands and fingers. After 1 year: dermatitis extended to face, upper part of the back, neck, elbows, and ankles (all parts air‐exposed). Lesions, acanthosis, spongiotic vesicles, lymphohistiocytes and eosinophils were present in the epidermis Allergic rhinitis		N/D; but dermatitis worsened after 1 year since firsts dermatitis.	Male; 37	Corticosteroid creams (class II)	^9^	2005
HATU HBTU	Chest tightness, cough and sneeze, and rashes (very red, sore, and itchy eyes and urticaria with angioedema affecting the exposed area of the face)		‐‐‐	Female; 29	‐‐‐	^10^	2003

Abbreviation: TBTU, 2‐[1*H*‐benzotriazol‐1‐yl]‐1,1,3,3‐tetramethyluronium tetrafluoroborate.

Despite using respiratory protection with a P2 filter (mask in accordance with standards DIN EN 143 and DIN 14387), a face shield, a fume hood, gloves, safety glasses, and a lab coat, these measures were insufficient to prevent contact with HBTU. Consequently, to continue working with peptides and their synthesis, F‐Res has explored alternative strategies, which do not involve uranium‐based coupling agent. She is now using Oxyma Pure as coupling agent with the Oxyma/DIC strategy.^12^, ^13^, ^14^, ^15^, ^16^, ^17^

Nowadays, reviewing the SDS documents associated with the different coupling agents, only the uronium peptide coupling agents (HBTU, HATU, and HCTU) report a statement regarding potential risk associated to its manipulation. For all these three coupling agents, it is reported a Skin Sens. 1A classification and the GSH hazard statement H317 (may cause an allergic skin reaction). Add more for HATU also “Resp. Sens. 1“ classification is reported in tandem with the GSH hazard statement H334 (may cause allergy or asthma symptoms or breathing difficulties if inhaled). Sub‐category 1A includes all substances that “showing a high frequency of occurrence in humans and/or high potency in animals can be presumed to have the potential to produce significant sensitization in humans.”

MSDS of Oxyma Pure does not report this information regarding potential health risks; however, that does not guarantee the absence of future sensitization. Herein, it is essential to handle it with all the precautions suggested for HBTU and maintain high attention on the onset of any potential symptoms susceptible to allergy and sensitization.

## 3 WE ARE THE ONES WHO MAKE THE CULTURE OF SAFETY

Every lab worker knows how essential it is to recognize, assess, and minimize or, if possible, eliminate risks when working in the laboratory. The recent development of the recognize, assess, minimize, prepare for emergencies (RAMP) paradigm^18^ offers a systematic approach to managing chemical hazards in the laboratory.

MSDSs or SDSs provide valuable chemical safety information, but sharing experiences regarding specific working situations of lab workers is crucial to avoid underestimating symptoms. This practice adds significant value beyond regulatory compliance and helps recognize safety incidents, even when symptoms occur hours after exposure.

The impact of this incident heightened awareness of the dangers associated with reagents that can induce sensitization over time, as well as the underestimation of initial symptoms in F‐Res's laboratory. F‐Res has shared her experience with co‐workers to minimize unintentional exposure risk to HBTU and to raise awareness about uronium peptide coupling agents.

Peptide coupling agents facilitate amide bond formation, which can modify human proteins. Therefore, anyone who comes into contact with a potential immune sensitizer should be vigilant for initial symptoms and handle the chemical with appropriate precautions, as with known sensitizers.

This experience taught everyone in F‐Res's lab to pay more attention to the use of MSDS documents and to seek safer reagents. The long‐term sensitization to HBTU, even in trace amounts in the air, and the potential longer term toxicological effects highlight the importance of safe laboratory practices and considering alternatives.

Sensitization should not be just a word in the Safety Data Sheet but an awareness taken seriously. Even if we may never become sensitized by a chemical, or know someone who is, the risk should never be neglected. Every lab worker should promote a culture of safety in the laboratory, and any occupational symptoms that develop should be communicated as widely as possible.

## AUTHOR CONTRIBUTIONS

The manuscript was written through contributions of the sole author.

## CONFLICT OF INTEREST STATEMENT

The author declares no competing financial interest.

## References

[1] El‐Faham A , Albericio F . Peptide coupling reagents, more than a letter soup. Chem Rev. 2011;111(11):6557‐6602. doi:10.1021/cr100048w 21866984

[2] Carpino LA , Imazumi H , El‐Faham A , et al. The uronium/guanidinium peptide coupling reagents: finally the true uronium salts. Angew Chem Int Ed. 2002;41(3):441‐445. doi:10.1002/1521-3773(20020201)41:3<441::AID-ANIE441>3.0.CO;2-N 12491372

[3] McKnelly KJ , Sokol W , Nowick JS . Anaphylaxis induced by peptide coupling agents: lessons learned from repeated exposure to HATU, HBTU, and HCTU. J Org Chem. 2020;85(3):1764‐1768. doi:10.1021/acs.joc.9b03280 31849224

[4] HBTU MSDS no. 12804 . Sigma‐Aldrich, Ed. 2023.

[5] Plackett B . What is an allergy sensitizer, and how does a chemical become one? ACS Chem Health Saf. 2020;27(2):75‐77. doi:10.1021/acs.chas.0c00025

[6] McAleer MA , Bourke B , Bourke J . Occupational allergic contact dermatitis to HBTU [(o‐bensotriazole‐10yl)‐N,N,N',N‐tetramethyluronium hexafluorophosphate]. Contact Dermatitis. 2010;62(2):123. doi:10.1111/j.1600-0536.2009.01685.x 20136901

[7] Vandenplas O , Hereng MP , Heymans J , et al. Respiratory and skin hypersensitivity reactions caused by a peptide coupling reagent. Occup Environ Med. 2008;65(10):715‐716. doi:10.1136/oem.2008.039123 18801928

[8] Hannu T , Alanko K , Keskinen H . Anaphylaxis and allergic contact urticaria from occupational airborne exposure to HBTU. Occup Med. 2006;56(6):430‐433. doi:10.1093/occmed/kql025 16861336

[9] Bousquet PJ , Guillot B , Guilhou JJ , Raison‐Peyron N . Occupational airborne allergic contact dermatitis due to HBTU. Contact Dermatitis. 2005;52(1):53‐54. doi:10.1111/j.0105-1873.2005.0483i.x 15701138

[10] Yung A , Papworth‐Smith J , Wilkinson SM . Occupational contact urticaria from the solid‐phase peptide synthesis coupling agents HATU and HBTU. Contact Dermatitis. 2003;49(2):108‐109. doi:10.1111/j.0105-1873.2003.0128h.x 14641366

[11] Miralles JC , Negro JM , Alonso JM , Garcia M , Sanchez‐Gascon F , Soriano J . Occupational rhinitis and bronchial asthma due to TBTU and HBTU sensitization. J Investig Allergol Clin Immunol. 2003;13(2):133‐134. 12968399

[12] Manne SR , El‐Faham A , de la Torre BG , Albericio F . Minimizing side reactions during amide formation using DIC and oxymapure in solid‐phase peptide synthesis. Tetrahedron Lett. 2021;85:153462. doi:10.1016/j.tetlet.2021.153462

[13] Albericio F , El‐Faham A . Choosing the right coupling reagent for peptides: a twenty‐five‐year journey. Org Process Res Dev. 2018;22(7):760‐772. doi:10.1021/acs.oprd.8b00159

[14] Collins JM , Porter KA , Singh SK , Vanier GS . High‐efficiency solid phase peptide synthesis (HE‐SPPS). Org Lett. 2014;16(3):940‐943. doi:10.1021/ol4036825 24456219

[15] Rich DH , Singh J . Chapter 5 ‐ The Carbodiimide Method. In: Gross E , Meienhofer J , eds. Major methods of peptide bond formation. Vol.1. Academic Press; 1979:241‐261. doi:10.1016/B978-0-12-304201-9.50011-4

[16] Subirós‐Funosas R , Prohens R , Barbas R , El‐Faham A , Albericio F . Oxyma: an efficient additive for peptide synthesis to replace the Benzotriazole‐based HOBt and HOAt with a lower risk of explosion[1]. Chem – Eur J. 2009;15(37):9394‐9403. doi:10.1002/chem.200900614 19575348

[17] Erny M , Lundqvist M , Rasmussen JH , Ludemann‐Hombourger O , Bihel F , Pawlas J . Minimizing HCN in DIC/Oxyma‐mediated amide bond‐forming reactions. Org Process Res Dev. 2020;24(7):1341‐1349. doi:10.1021/acs.oprd.0c00227

[18] Finster DC . RAMP: a safety tool for chemists and chemistry students. J Chem Educ. 2021;98(1):19‐24. doi:10.1021/acs.jchemed.0c00142

